# *Salmonella* and Salmonellosis in Wild Birds

**DOI:** 10.3390/ani14233533

**Published:** 2024-12-06

**Authors:** Paul Wigley

**Affiliations:** Bristol Veterinary School, University of Bristol, Bristol B40 5DU, UK; paul.wigley@bristol.ac.uk

**Keywords:** *Salmonella*, zoonoses, avian, raptor, *Anseriformes*, passerine, *Columbiformes*, systemic infection, pathogen genomics, host adaptation, disease transmission

## Abstract

*Salmonella enterica* is an important bacterial pathogen in humans and warm-blooded animals. Wild bird species represent both a potential reservoir for zoonotic infection and as a susceptible host to infection by host-adapted variants. Historically, wild birds were considered to be a major source of *Salmonella* infection in livestock, but in recent years, it has been more apparent that birds are more likely to act as a reservoir for recycling infection on farms rather than as the primary source of infection. Birds may also transmit infection to humans directly from feces or indirectly through fecal contamination of foods, including peanut butter. While many bird species can be infected with *Salmonella*, the rates of infection are variable, and most cases lead to intestinal carriage rather than disease. In this case, fecal shedding of *Salmonella* bacteria from birds can represent a risk for transmission to humans. As such, care is needed when in contact with fecal material such as that found on bird tables or feeders. In recent years, there have been emergences of *Salmonella* Typhimurium genotypes associated with high mortality in songbirds or passerine birds, resulting in ‘die offs’ in Europe, Israel, New Zealand and the US. Additionally, *S. typhimurium* DT2 and other variant Copenhagen genotypes are associated with high mortality disease in pigeons. These genotypes show evidence of evolution towards adaptation to specific hosts, with pseudogenes leading to loss of functional metabolic pathways and specific virulence factors. These ‘signatures of adaptation’ are common in host-adapted *Salmonella* serovars and suggest these *S. typhimurium* isolates are evolving to adapt to specific avian hosts.

## Simple Summary

Wild bird species are known to be readily infected with and carry *Salmonella enterica*. This has two main consequences. Firstly, they may act as reservoirs in the transmission of infection to livestock species or zoonotic transmission to humans. Secondly, birds themselves may develop systemic infection, leading to high mortality disease. Recent studies have shown that specific genotypes adapted to and capable of causing disease in a range of bird species have recently evolved.

## 1. Introduction

While the Gram negative bacterial species *Salmonella enterica* is most widely known as a foodborne pathogen, it is also a major animal pathogen leading to gastroenteritis, including *Salmonella enterica* serovar Typhimurium scouring in calves [1] and to invasive diarrheal disease in dogs [2]. The species is made up of more than 2500 serovars or serotypes based on serological typing of flagella (H) and lipopolysaccharide (O) antigens. Most serovars are associated with intestinal infection, but host-adapted and host-restricted serovars can lead to systemic or typhoidal-like disease in their host, including *Salmonella enterica* serovar Cholerasuis in pigs [3], *Salmonella enterica* serovar Dublin in cattle [4] and *Salmonella enterica* serovar Gallinarum, the cause of fowl typhoid in galliform birds [5]. Finally, serovars Abortusovis and Abortusequi are responsible for abortion in sheep and goats, and equid species, respectively [6].

Wildlife and, in particular, wild birds are not immune to salmonellosis, and infection may result in severe disease, which may manifest as ‘die-offs’. Additionally, wild birds are considered a major vehicle of *Salmonella* infection in production animals and even a direct source of *Salmonella* entry into certain foodstuffs. In this review, we will consider the role wild birds play in the transmission of *Salmonella,* salmonellosis as a disease of wild birds and, finally, how genome-based approaches are allowing us to understand the evolution of *Salmonella* as a pathogen of wild bird species and even the host response to infection.

## 2. Wild Birds and Zoonotic *Salmonella* Transmission

Zoonotic transmission of *Salmonella* can involve wild birds both directly from feces and from the direct fecal contamination of produce or processed foods. It can also play an indirect role in the infection of food-producing animals. These potential routes are discussed below:

### 2.1. Wild Birds as Vectors of Transmission into Livestock

Wild birds and rodents have long been considered to be a major source of pathogen entry into livestock production [7], and control measures are frequently applied to housed animals such as pigs and broiler chickens to prevent ingress of wild birds. Typically, studies have relied on cultures of *Salmonella* from fecal samples or captured wild birds around livestock production to suggest it as a source of infection [8,9,10]. A scoping review conducted by Grieg and colleagues in 2015 [11] indicated close to 300 studies that identified an association between *Salmonella*, wildlife and livestock but noted that few studies had employed any molecular epidemiological tools to ascertain the relationship of *Salmonella* isolated or the direction of transmission. Indeed, some have questioned the perception that wild birds are a major source of *Salmonella*, given its capacity to cause disease in wild birds and the low prevalence of *Salmonella* without disease (6.4% in US studies), with an inherent bias of sampling towards birds such as starlings, sparrows and gulls, all of which have a closer association with people [12]. This is supported by UK studies, which showed that *Salmonella* was found in under 1% of healthy passerine birds, and that isolates found in dead birds were frequently distinct from those seen in livestock [13]. More recent studies suggest that the epidemiological role of wild birds in livestock infection is to act a reservoir of the bacterium, facilitating the maintenance of infection on farms, rather than a primary infection source [14,15]. This is perhaps best illustrated by an elegant study on the role of wild birds in outdoor pig production in the UK birds [16]. The pig-associated DT193 monophasic *S. typhimurium* genotype was found in wild bird droppings after fields were used by breeding pigs, suggesting the route of transmission is pig to bird, but the prevalence of *Salmonella* continued to rise in bird droppings for several years after the pigs were removed, indicating a role for persistence and ongoing transmission, even if not the primary source of infection.

### 2.2. Wild Birds and Food Contamination

Direct fecal contamination of fruit and vegetables can lead to *Salmonella* infections regardless of source, including livestock, wildlife and human feces [17]. A recent study has shown a high prevalence of *Salmonella* in wild bird feces associated with fresh produce, highlighting this risk [18]. Contamination of certain processed foods, most notably chocolate and peanut butter, has implicated wild bird feces as a potential source. *Salmonella* is able to survive well in these high-fat, low-water-availability products and show increased thermotolerance in such food matrices [19]. Contamination may be of raw ingredients but more likely the processed or partly processed product, which has been considered the source of peanut butter contamination in the US and which led to a large national outbreak of *Salmonella enterica* serovar Tennessee [20]. Contaminated water dripping from the factory roof, with the likely source of this being wild bird droppings, was considered and definitively proven as the source of *Salmonella* [21]. Chocolate contaminated by wild bird feces was shown to be the source of *S. typhimurium* in an outbreak in Norway [22,23], with several subsequent outbreaks being, at least anecdotally, associated with birds.

### 2.3. Direct Human Infection from Wild Birds

*Salmonella* infection may also occur due to direct exposure to both domestic and wild birds and their feces. In recent years, there has been an increase in awareness of backyard poultry as a source of infection [24], but transmission from wild birds is also a public health issue, often through fecal contamination of garden feeders and bird tables [25]. As such, it is recommended that good hygiene is practiced when cleaning or filling bird tables and feeders and that vulnerable individuals avoid undertaking this.

## 3. Salmonellosis in Wild Birds

Broadly, *Salmonella* infections of wild birds can range from colonization of the gut with no overt disease to severe systemic infections seen in ‘die offs’ of pigeons and passerine birds [26,27]. In theory, all birds could be colonized by an enormous range of serovars, and, indeed, experimental infection of chickens has been seen with many serovars [28,29]. An extensive review by Tizard in 2004 details *Salmonella* infections and related pathologies in a range of avian groups, including gulls, raptors, pigeons, songbirds and waterfowl [30]. While there is little new to add to our understanding of the pathology resulting from salmonellosis and the species affected by it, here, we aim to update these findings and detail our increased understanding of host and pathogen biology.

### 3.1. Range of Species Infected with Salmonella

Gulls are considered to bear a major risk of carriage of both *Salmonella* and another major foodborne pathogen, *Campylobacter*, due to their scavenging and feeding off human refuse, though studies show wide variation in carriage, from fewer than 3% of black-headed gulls sampled in Sweden [31] to more than 50% of the same species in the Czech Republic [32]. As gulls move around multiple human-made landscapes, they are considered to represent a specific risk in both direct and indirect pathogen transmission [33]. Gulls are considered a nuisance in some urban areas due to their aggressive behavior towards humans [34] and a clear route to direct transmission via feces. Indirect transmission may also occur, with gulls considered the reservoir of *S.* Newport contaminating tomatoes via feces in the USA [35].

Pigeons are susceptible to systemic disease caused by an adapted variant of *S. typhimurium*, though they may also carry other strains. *S. typhimurium* variant Copenhagen was first described more than fifty years ago as the cause of paratyphoid disease in pigeons [36]. Comprised of DT2 and DT99 phage types, this pathovariant is associated with high virulence, including hemorrhagic diarrhea, hepatosplenomegaly and granulomatous legions of the liver, which can be reproduced experimentally in pigeons with increased pathology over ‘standard’ isolates from mammalian hosts [37]. Paratyphoid disease is a considerable worry to pigeon ‘fanciers’, who breed and race pigeons to an extent that vaccines are frequently used in domesticated pigeons.

Both captive and wild raptors as well as scavengers such as vultures have been shown to carry a range of *Salmonella* serovars [38,39,40]. For example, a Spanish study found *Salmonella* in around 4% of fecal samples from wild raptors, including serovars Enteritidis, Adelaide, Brandenburg, Newport, Typhimurium, Hadar, Saintpaul and Virchow [41]. *Salmonella* also appears to be more frequent in captive birds, potentially from food sources such as captive bred rodents or chicks [41,42,43]. While most carriage in raptor species represents a limited threat to bird health, in captive birds, there can be spreading in rehabilitation centers [42], and infection remains a significant threat to susceptible newly hatched chicks [44]. One recent study showed a high prevalence of multi-drug-resistant monophasic *S. typhimurium*-like strains that are predominant in pig production in Europe in raptors at a wildlife recovery center [45]. While this may imply a role for raptors as a reservoir for infection in pigs, it is unclear if the birds were captive fed prior to sampling. As raptors have limited human contact, their role in transmission to humans is not considered significant. However, a curious outbreak of *S. typhimurium* occurred following contamination of cafeteria tables by a school science club [46]. The club had been dissecting owl pellets on the tables, which led to contamination following inadequate cleaning and disinfection, leading to illness in several students subsequently using the cafeteria.

Both wild and domesticated waterfowl can harbor *Salmonella*. Although not as well studied as chickens, commercial duck meat and duck egg production are a significant, though often overlooked, source of human salmonellosis, with large outbreaks of *S. typhimurium* DT8 in the UK and Ireland between 2009 and 2011 [47,48], and many studies of *Salmonella* in ducks along the food chain in large scale commercial and backyard production highlight this potential risk [49]. The prevalence of *Salmonella* in wild waterfowl varies across studies. Free-living Canada geese sampled in Canada were found to have a *Salmonella* prevalence of around 11% [50], and waterfowl sampled from ponds in parks in Ohio had a prevalence of 50% [51], suggesting waterfowl may represent a potential reservoir for zoonotic transmission. In contrast, large studies in the Texas Gulf Coast area found *Salmonella* in only two of close to 400 waterfowl sampled (prevalence 0.5%) [52]; in six of 600 bird samples in Argentina, a prevalence of 1%, [53] and was not detected in over 300 adult waterfowl in northern Spain [54], suggesting that free-living waterfowl represent a low risk of zoonotic transmission.

Perhaps the most visible manifestation of salmonellosis in wild birds is in passerine or songbird species, where *Salmonella* infection has been implicated in high mortality ‘die offs’ of sparrows and finches. In the UK, the work of Pennycott described the disseminated infection and pathology associated with *S. typhimurium* in sparrows and finches, which in retrospect led to an epidemic of ‘die offs’ between 2005 and 2012 [55,56,57,58]. This work identified the house sparrow (*Passer domesticus*) and greenfinch (*Carduelis chloris*) as particularly affected, noted the association of specific *S. typhimurium* phage types DT40 and DT56var and noted that mortality is primarily in winter and is associated with secondary feeding in gardens via feeders and bird tables of these gregarious and social species. More detailed studies in northern England showed disseminated pathology characterized by necrotic lesions in dead passerines where *S. typhimurium* was cultured, though fecal samples of healthy birds captured by mist netting showed low carriage of *Salmonella* in these species [13]. Furthermore, molecular typing identified that many birds were infected with a clonal *S. typhimurium* DT56 var., which was subsequently found to have the unique genotype ST568 [59]. *Salmonella* isolates in these studies were also notable in being almost entirely susceptible to antimicrobials, indicative of infection circulating in a population and not being under the selection pressure of livestock where antimicrobial resistance is common.

In New Zealand, a prolonged outbreak of *S. typhimurium* DT160 from 1998 to 2012 led to both high mortality in garden birds and human enteritis, with particularly high mortality in sparrows early in this outbreak [60,61,62,63]. Genomic analysis revealed that the original DT160 became more varied and remained associated with multiple hosts. Although routes of transmission between wild birds, humans and livestock could not be defined, there is a degree of consensus that wild birds were the original source and the main vehicle of at least the earlier human cases [60]. In the UK, the phage types associated with wild birds and shared with humans represented only 0.2% of total human *Salmonella* cases, which suggests a potential, if relatively small, role as a reservoir for human disease [25].

Studies in Israel in a range of sparrow species have shown a unique monophasic *S. typhimurium* type, which, unlike most *S. typhimurium*, do not switch flagella type (phase variation) [64]. Phase variation is thought to offer an advantage in overcoming host immunity, though studies of monophasic variants of *S. typhimurium* suggest that monophasic strains are not disadvantaged in cell-based or in vivo infection models, nor are naturally monophasic serovars such as *S.* Enteritidis in anyway disadvantaged [65,66], though this variant differs from the epidemic strains found in pigs across Europe. A unique new serovar, termed *S.* Tirat-Zvi and associated with sparrows, has also been found in Israel [67].

### 3.2. Adaptation of S. typhimurium to Avian Hosts

In the last 25 years, it has become apparent that there is a strong association between specific *S. typhimurium* strains and specific types of wild birds. While most *S. typhimurium* variants sequenced belong to the ST19 genotype, there are a number of genotypes associated with specific hosts and frequently with more invasive disease [68]. This adaptation can leave clues in the genome or ‘signatures of adaptation’. In *Salmonella*, these typically include shrinkage of the function genome with loss of genes, genomic inversions and pseudogenes being formed, the latter two leading to loss of function if not actual genome size. Pseudogenes are non-functional stretches of DNA that resemble a functional gene. They are common in eukaryotes, where they often called ‘junk DNA’, but unusual in bacteria. The presence of pseudogenes in bacteria is a useful tool in understanding evolution, as they give a picture of pathways and proteins that are no longer needed after adaptation to a specific host or environment. In the host-restricted serovar *S. gallinarum*, both a loss of genome size and up to 300 pseudogenes have been identified in the genome [69]. In the main, these account for the loss of metabolic pathways involved in gut colonization or adhesins, such as fimbriae, involved in gut attachment.

**Figure 1:**
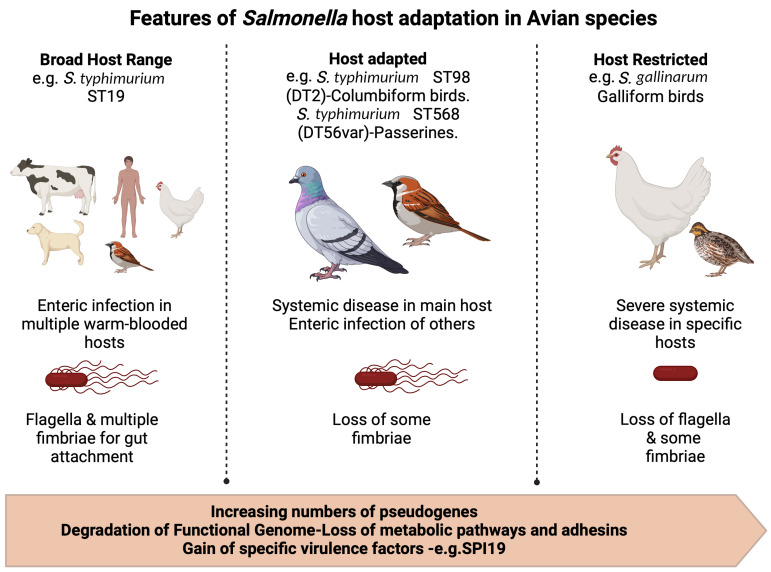
The major genomic and phenotypic changes associated with host adaptation of *Salmonella* in bird species. Created in BioRender. Wigley, P. (2024) https://BioRender.com/u38k260.

While the changes in host-adapted *S. typhimurium* are less marked, genomic comparison between the ‘classical’ *S. typhimurium* strain SL1344 (ST19) and the DT2 strain 94–213 (ST98) revealed an additional 21 pseudogenes in a range of structural and metabolic associated proteins [26]. Furthermore, at 41 °C, the core body temperature of a pigeon, there was a differential expression of a range of genes largely associated with motility, indicative of DT2’s adaptation to its host. Genomic analysis of UK passerine strains in comparison to human strains and other animal strains of the same phage type revealed little difference but defined ST568 as a distinct genome clade [27]. More recent comparisons of US and UK passerine isolates have shown the recent evolution of these strains from gulls within the last 200 years [70]. Moreover, this study also showed an accumulation of pseudogenes in virulence factors, including fimbriae involved in attachment to host epithelium and type III secretion systems that translocate effector proteins or toxins to host cells that are associated with enteric disease and colonization of the gut. This is further reinforced by work on isolates associated with systemic disease in sparrows in Israel, where genome sequencing showed a close relationship to the British ST568 strains [64]. The four Israeli strains also showed an accumulation of pseudogenes in both type III secretion systems and in adhesins, including fimbriae, but perhaps just as significantly in metabolic pathways, including tetrathionate metabolism. All three studies showed that most passerine isolates do not carry *Salmonella* virulence plasmids and are generally susceptible to antimicrobials. Taken together, these point towards an ongoing evolutionary adaptation to causing systemic infection in a specific or narrow range of hosts, leaving evidence as ‘signatures of adaptation’ in the genome. This is also supported by evidence of distinct evolutionary clades within different bird types, with songbirds, gulls and wading fowl each hosting their ‘own’ *S. typhimurium* [71]. It would also seem that passerine-adapted strains have evolved separately in North America, Europe and New Zealand, though the close relatedness of these clades suggests a likely common ancestor [70]. The basis of the likely host adaptation by *Salmonella enterica* is summarized in Figure 1.

## 4. The Host Response to Infection

Most of our understanding of the immune response to salmonellosis in birds comes from chickens and other domestic species. The roles of innate, humoral and cellular adaptive responses and genetic resistance to disease have all been extensively studied [72]. It would seem likely that similar mechanisms are likely in wild birds, though defining these is extremely difficult. There are limited studies of immune function in captive passerines, including *Salmonella* challenge experiments which are indicative of epigenetic differences of innate immune function impacting the outcome following infection [73]. It is also tempting to speculate that factors such as pollution and pesticides in the environment may be having detrimental effects on immune function and increasing susceptibility in wild birds, which may have contributed to ‘die offs’.

## 5. Conclusions

*Salmonella enterica* is an important infection of wild birds both in terms of disease and of potential zoonotic transmission. While recent epidemiological evidence suggests that birds are not the main source of primary infection of livestock with *Salmonella*, they remain a key reservoir in its maintenance on farms. Direct or indirect contact with the feces of infected birds remains a risk. As such, it is important to control the entry of wild birds into food production areas and to exercise good hygiene practice when feeding birds or cleaning feeders and bird tables. Messages to the public around this need to be made clearer both in terms of the zoonotic and epizootic risks of transmission around contaminated feeders and tables.

The advent of molecular epidemiology and pathogen genomics has revealed the emergence of several genotypes of *S. typhimurium* associated with high mortality systemic infections in a range of bird species. These genotypes show distinct genomic ‘signatures of adaptation’, indicating a process of evolution taking place to adapt to these species. Together with increased transmission risk due to anthropogenic feeding of birds, the emergence of these genotypes may contribute to the observed mass mortality events or ‘die offs’ seen in songbirds in recent times.

As whole genome sequencing (WGS) becomes more commonplace, we are likely to see more evidence of adaptation across avian species and *Salmonella* serovars. Perhaps it was inevitable that these changes were seen in birds more closely associated with human activity, like the domesticated chicken, the urban pigeon and garden songbirds.

There are clearly huge gaps in our knowledge around transmission, the wider population of *Salmonella* within the wider population of bird species and the role of factors such as migration in pathogen dissemination, as is seen for influenza.

As WGS becomes the norm in the public health investigation of salmonellosis cases in humans, it should also lead to a clearer understanding of the risk of zoonotic *Salmonella* transmission from wild birds, though this will likely be dwarfed by the risks arising from food consumption of animal products.

## Data Availability

No new data were created or analyzed in this study.
